# Regularizing hyperparameters of interacting neural signals in the mouse cortex reflect states of arousal

**DOI:** 10.1371/journal.pcbi.1012478

**Published:** 2024-10-15

**Authors:** Dmitry R. Lyamzin, Andrea Alamia, Mohammad Abdolrahmani, Ryo Aoki, Andrea Benucci

**Affiliations:** 1 RIKEN Center for Brain Science, Wako-shi, Saitama, Japan; 2 Centre de Recherche Cerveau et Cognition, CNRS, Université de Toulouse, Toulouse, France; 3 Queen Mary University of London, School of Biological and Behavioural Sciences, London, United Kingdom; Brain and Spine Institute (ICM), FRANCE

## Abstract

In natural behaviors, multiple neural signals simultaneously drive activation across overlapping brain networks. Due to limitations in the amount of data that can be acquired in common experimental designs, the determination of these interactions is commonly inferred via modeling approaches, which reduce overfitting by finding appropriate regularizing hyperparameters. However, it is unclear whether these hyperparameters can also be related to any aspect of the underlying biological phenomena and help interpret them. We applied a state-of-the-art regularization procedure—automatic locality determination—to interacting neural activations in the mouse posterior cortex associated with movements of the body and eyes. As expected, regularization significantly improved the determination and interpretability of the response interactions. However, regularizing hyperparameters also changed considerably, and seemingly unpredictably, from animal to animal. We found that these variations were not random; rather, they correlated with the variability in visually evoked responses and with the variability in the state of arousal of the animals measured by pupillometry—both pieces of information that were not included in the modeling framework. These observations could be generalized to another commonly used—but potentially less informative—regularization method, ridge regression. Our findings demonstrate that optimal model hyperparameters can be discovery tools that are informative of factors not a priori included in the model’s design.

## Author summary

Statistical and machine learning models are increasingly being utilized to analyze and interpret neural data, often in conjunction with statistical regularization methods to improve the accuracy and robustness of the models. Here, we demonstrate that regularization techniques can be used to gain insights into neurophysiological phenomena which are not pre-specified in the original modeling framework. This was demonstrated by modeling cortical neural activations in the mouse posterior cortex during a visual discrimination task. Application of a state-of-the-art regularization technique, automatic locality determination (ALD), revealed that the optimized hyperparameters varied irregularly among mice. However, when correlating this variability with neural and behavioral data not initially provided to the model, such as visual-evoked responses and pupil dilations, we observed that the variance in hyperparameters was indicative of across-animal differences in average states of arousal. Additionally, we showed that these conclusions were not limited to ALD, but were also applicable to a simpler regularization technique, ridge regression. Our findings suggest that regularization hyperparameters can be utilized as valuable discovery tools and should be employed more frequently for data interpretation alongside model parameters.

## Introduction

During natural behaviors, sensory-motor and internal variables (e.g., decisional and attentive) interact across overlapping brain networks [1–4]. Understanding the properties of these interactions is critical to shed light on the underlying computations; for instance, nonlinear mixing of task-related variables can be informative of the dimensionality of the neural representations and, accordingly, of the set of input–output transformations achievable by the network [4]. Interactions between variables can also be related to the classification invariances learned by a network [5], inference and predictive coding computations [3, 6–8], as well as context-dependent processes [2].

A common and interpretable approach to modeling the interaction dynamics between two or more variables is by using a series expansion, which is defined as the sum of the activations of individual variables (1^st^ order terms or “kernels”) and higher-order terms for the interactions between the variables (2^nd^ and higher-order kernels) [9–12]. Even when the expansion is stopped at 2^nd^ order terms (e.g., [13]), the number of parameters needed to estimate the associated kernels grows quadratically with the duration of the interaction. This is because two-dimensional temporal kernels define the amplitude of the interaction at all pairs of time lags relative to the timing of the individual variables. In other words, a 2^nd^ order interaction term is defined as a “square” in the temporal domain, and each point in the square defines a specific temporal relationship between the two variables. The precise determination of these kernels is therefore challenged by the large amount of data needed to estimate all pairs of time lags, aggravated by a “combinatorial explosion” in parameter estimation for higher-order interaction kernels, which typically leads to overfitting.

To overcome this data size limitation, modeling approaches using adequate regularization are commonly employed to estimate these kernels from incomplete data representations [14]. The hyperparameters that control the regularization process often have a straightforward mathematical interpretation; they constrain the solutions of the statistical model and improve its predictive power. However, the relationship between these hyperparameters and the underlying physiological processes remains unclear: Do these hyperparameters merely improve model fitting, or can they be related to the underlying biological phenomena and help interpret them?

We addressed this question in the mouse sensory-motor system and in the context of the interactions between well-documented movement-related neural activations associated with movements of the eyes and of the body [13]. These activations are not unique to mice: in all mammals, movement-related signals modulate the activity of large brain networks together with sensory inputs [15–18]. In the visual system, these broadly distributed signals can significantly influence activations, even in the primary visual cortex, as described in primates [19] and very extensively in rodents [13, 20–22]. Given that movement-related signals typically occur in complex sequences, they generate activations that temporally overlap across large brain networks [13]. In mice, we and other groups have shown that movements potently activate the primary and higher visual cortices [13, 22], and in previous work [13], we used a generalized linear model to determine the time-dependent response interactions between movement-related signals (defined as quadratic, two-dimensional temporal kernels) from widefield recordings (GCaMP6s) as animals engaged in visual discrimination tasks. In particular, in wide-field fluorescence imaging, the signal from each camera pixel reflects calcium-related activity in large ensembles of neurons from superficial cortical layers, as well as dendritic activations of cells with somas in deeper layers [23]. These signals are relatively slow (e.g., compared to spiking dynamics) and exhibit significant spatial correlations.

Here, we applied regularization techniques to this modeling approach and examined the associated regularizing hyperparameters in the broader context of behaviorally relevant task variables. Specifically, we adopted a regularization technique exemplifying recent state-of-the-art methods based on Bayesian priors—automatic locality determination (ALD) [24]. The key idea in ALD is to estimate a few hyperparameters determining the position and shape of priors, which define a contiguous region in the temporal and frequency domain of the kernel parameters space. When applied to the interacting movement-related responses, ALD priors identified a compact region of the interaction kernel with negative (“suppressive”) parameters, in line with our previous findings describing the sub-linear summation between signals evoked by eye and body movements [13]. In the frequency domain, the ALD hyperparameters varied across animals, along with the strength of the suppression in the interaction kernels. This variability was not random but could be linked to the differences in the trial-to-trial variability of the visually evoked responses between mice. Notably, this variability could be attributed to differences in the animals’ arousal levels, which we gauged from changes in pupil diameter, which, in turn, correlated with variations in ALD frequency hyperparameters. A similar relationship between regularizing hyperparameters and arousal levels could also be observed—qualitatively—using the more conventional regularization technique—Ridge (L2) regularization—providing evidence for the generality of our conclusions relative to the specifics of the regularization procedure.

Our results demonstrate that regularization techniques not only improve model fitting but may also aid in the interpretation of the data, revealing phenomena in the underlying physiological processes not included a priori in the original model design.

## Results

### Behavioral task, neural responses, and generalized linear model

**Fig 1:**
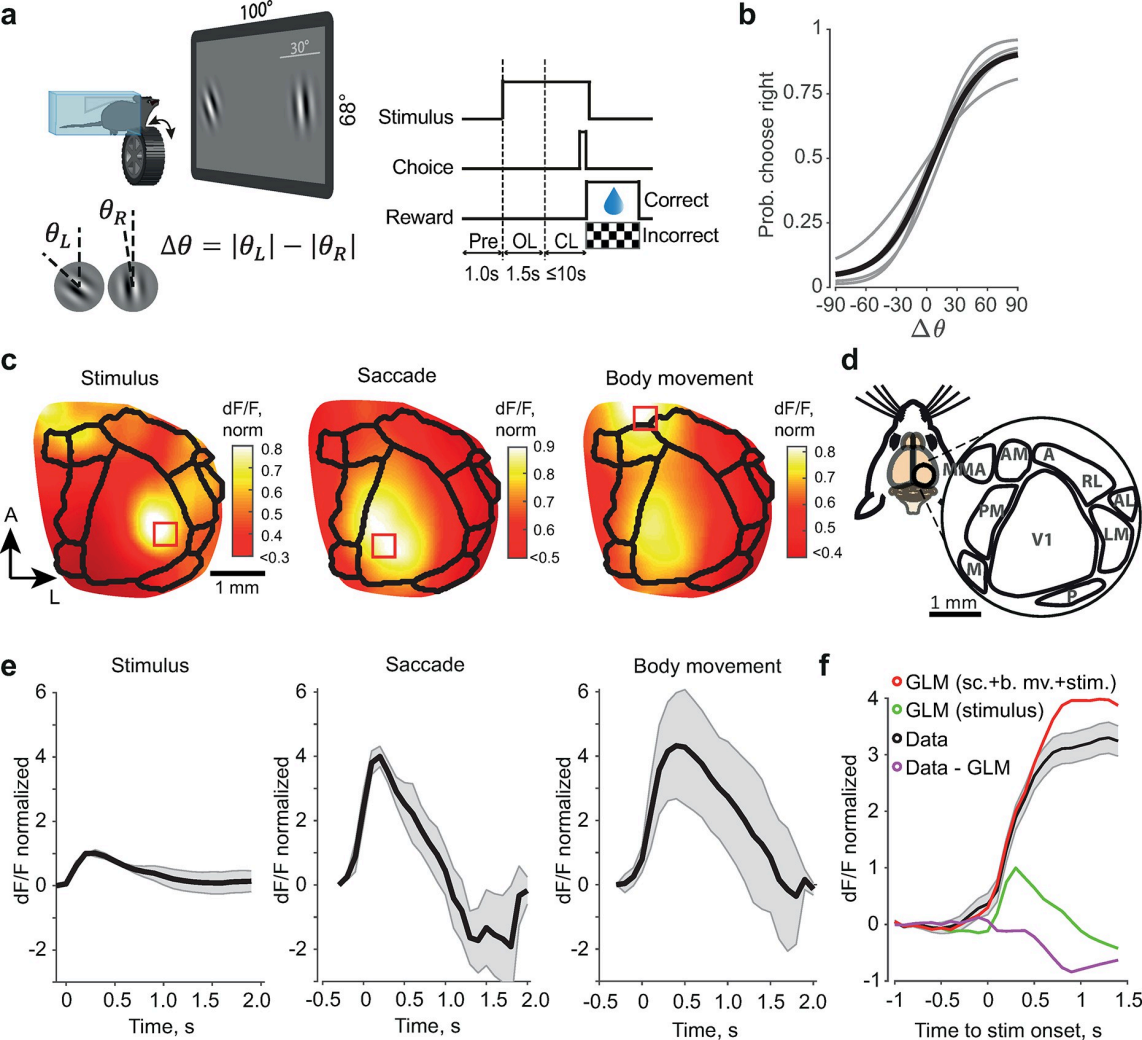
Experimental design, behavioral performance, neural recordings, and first-order GLM. (**a**) Left, top: Experimental setup. The animal moves both targets on the screen using the wheel manipulator, and is rewarded for placing the more vertically oriented stimulus in the center of the screen. Left, bottom: orientation angles are computed relative to the vertical (“+” clockwise, “-”counter clockwise), task difficulty Δ*θ* is measured as a difference in verticalities |*θ*_*L*_|−|*θ*_*R*_|. Right: Phases of a trial. Pre: pre-stimulus period; OL: open loop in which the wheel does not move the stimuli; CL: closed loop in which the wheel moves the stimuli. (**b**) Probability of making a right-side choice as a function of task difficulty Δ*θ*. Black line–population average; gray lines–individual mice. (**c**) Population average of peak-scaled GCaMP responses to visual stimulus (left), saccade (center), and body movement (right). The black lines show retinotopically identified visual areas (see (d)). The red squares indicate the representative animal’s regions of interest for the dF/F time traces in (e). The arrows indicate the anterior (A) and lateral (L) directions. (**d**) Location of the imaged part of the brain (left), and retinotopically defined visual areas (right): primary visual cortex (V1), lateromedial area (LM), rostrolateral area (RL), anterior area (A), anteromedial area (AM), medial area (M), posteromedial area (PM), posterior area (P), anterolateral area (AL), and mediomedial area (MMA). (**e**) Stimulus, saccade, and body movement responses normalized to the amplitudes of the response to the visual stimulus at its peak time and averaged across the regions of interest of individual mice (means ± SE), red squares shown in (c). (**f**) Example animal, trial average fluorescence from the stimulus ROI shown in (c), left panel, aligned to the stimulus onset (black, error bars), average visual response predicted by the GLM (green, only from trails with no detected saccade or body movement), average prediction of the GLM with stimulus, saccade, and body movement responses included (“first-order GLM,” red; the times of saccades and body movement change from trial to trial), and residual between the data and first-order GLM prediction (purple). All amplitudes are normalized to the peak amplitude of the response to the visual stimulus.

We used an automated setup featuring voluntary head fixation [25] to train transgenic mice (Thy1-GCaMP6f; n = 5) in a two-alternative forced choice (2AFC) orientation discrimination task (***Fig 1A and 1B***). The animals chose the more vertical of two simultaneously presented Gabor gratings [26] and indicated their choices by rotating a wheel manipulator. The correct responses were rewarded with 4 μl of water. After the animals reached a success rate of 75%, we recorded the neural activity during the task in their posterior cortices using wide-field imaging of the GCaMP signals. We also recorded the position of the pupils, from which we determined eye movements (saccades; see Materials and Methods). From the position of the wheel manipulator, we determined the wheel rotation onset times. The wheel rotations were associated with broader movements of the whole body, including the trunk, hind limbs, and tail; hence, we refer to them as body movements. The data for each trial consisted of cortical fluorescence video frames, binary vectors of eye and body movements [13], and a binary vector representing the stimulus onset time (fixed across trials). In all analyses, we considered data only from 1 s before to 1.5 s after stimulus onset, during which the stimulus did not move on the screen (***Fig 1A***; see Materials and Methods).

All three event types—body movements, saccades, and stimulus onsets—were associated with cortical activations. Neural activations in response to stimulus onsets were strongest in the primary visual cortex, V1 (***Fig 1C***, left; ***Fig 1D***). Responses to saccades and body movements were broadly distributed across the cortex and did not follow the boundaries of retinotopically identified higher visual areas [13]. Saccade responses reached their maximum amplitudes near the medial edge of V1, while the strongest body movements were detected in the same region and in the region anterior to area AM (***Fig 1C***, center, right; ***Fig 1D***). In all analyses, we report GCaMP activity uncorrected for a hemodynamic component, as control experiments confirmed that such a component was small in the visual cortex, and in the temporal windows considered in our analysis, hemodynamic corrections were approximately one order of magnitude smaller than the main reported effects during movement interactions. (***S1 Fig***) and localized in the proximity of blood vessels [27].

We modeled the GCaMP responses using a generalized linear model (GLM) with an identity link function and saccade, movement, and stimulus event vectors used as inputs, in each region of interest (ROI) separately (***Fig 1C***), according to the equation $y(t)=\sum_{i=1}^{I}w_{i}*x_{i}+\varepsilon$, where *w*_*i*_ represent the convolution kernels, *ε* is Gaussian noise, and *x*_*i*_ are the event vectors of inputs. In the following, OLS kernels were computed using the normal equation $\hat{w}={\left(X^{T}X\right)}^{-1}X^{T}y$, where *X* is a design matrix of *x* with rows corresponding to time bins of *y* and columns corresponding to kernel dimensions *i*.

First, we examined the neural responses to each of the three events in isolation. We selected trials with no saccades or body movements within the time interval of interest and fitted a stimulus-evoked GLM kernel using these trials only (***Fig 1C and 1E***, left). We then subtracted the estimated stimulus response in trials with either eye or body movements and used residuals to compute the saccade and body movement kernels. We fitted the wheel movement and saccade kernels within a -0.3 to 2-s interval around the respective event using ridge-regularized least squares (***Fig 1C and 1E***, center, right), with the regularization hyperparameter found automatically through evidence maximization [24]. Using the estimated response kernels, we predicted GCaMP responses in all trials, including trials with many co-occurring saccades and body movements and in all ROIs. Out of a mean of 2965 ± 353 trials per animal, 567 ± 53 trials had these co-occurring events, with 2.04 ± 0.11 saccade–body movement pairs per trial. As evidenced by the trial time averages (***Fig 1F***), the GLM predictions overestimated the neural responses and resulted in negative residuals. We obtained an adequate estimate of isolated saccades and body movements, and, having shown before that the GCaMP signal was not saturated in this regime [13], we inferred that these negative residuals were due to suppressive nonlinear interactions between co-occurring saccades and body movements.

### Interaction kernels

**Fig 2:**
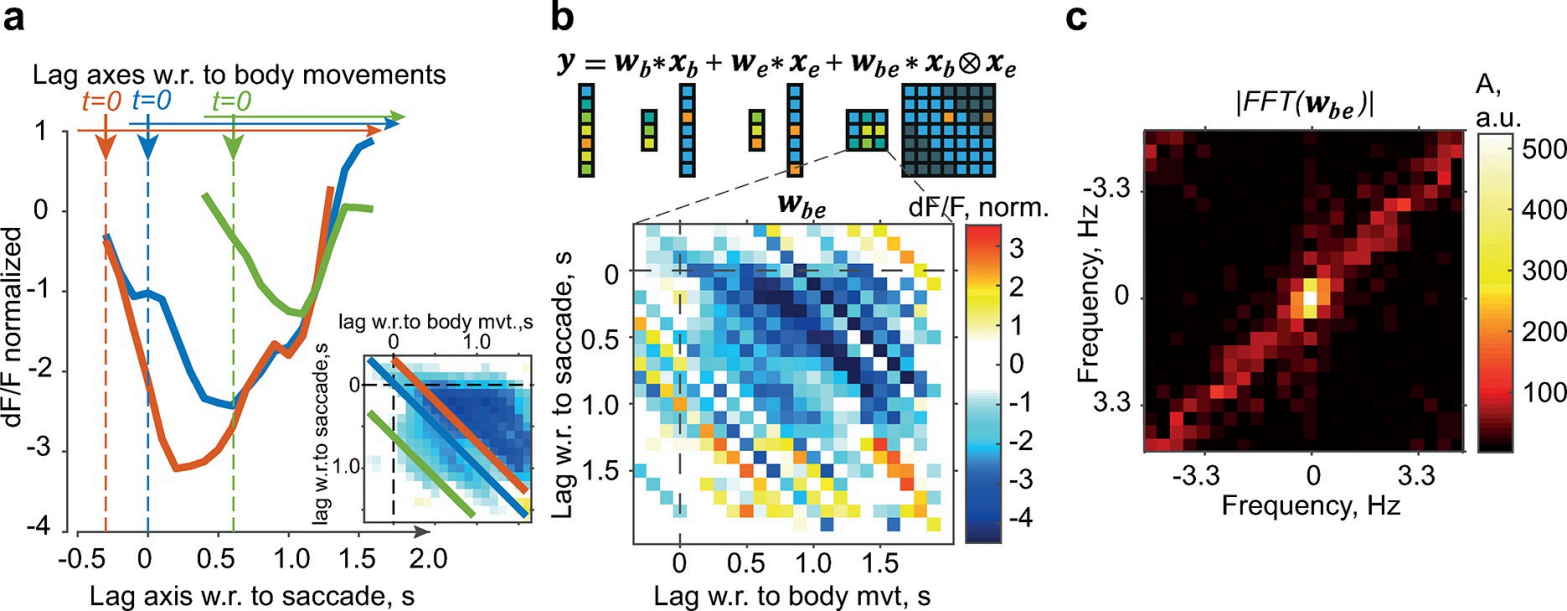
Interactions of saccade and body movement responses modeled using second-order GLM kernels. (**a**) Average dF/F residuals after subtracting stimulus, saccade, and body movement responses in trials with co-occurring saccades and body movements for three examples of relative timing between body movement and saccade. Bottom horizontal axis: time lag relative to the saccade. For all three curves, a saccade occurs at 0 s. Top horizontal axis: time lag relative to body movement onset; three different t = 0 positions relative to the saccade axis. Inset: interaction kernel that captures residual values for all possible relative timings of saccades and body movements within a time interval (-0.3 s, 1.7 s) around each event. Residuals on the left correspond to three diagonals of the same color; thus, a diagonal corresponds to a fixed relative lag (relative timing) between a saccade and a body movement. (**b**) Top: Schematic of the second-order GLM expansion for saccades (subscript “e,” eye movements) and body movements (“b”). The response is modeled as the sum of eye and body movement inputs (event vectors x_b_, x_e_) convolved with respective linear kernels (w_b_, w_e_) and an interaction kernel w_be_ convolved with a two-dimensional eye–body movement input (x_b_ ⊗ x_e_). Replotted from ref. [13]. Bottom: Ridge regression estimate of the interaction kernel; example animal. dF/F is normalized to the stimulus response amplitude. (**c**) Absolute value of the two-dimensional Fourier transform of the kernel in (**b**), centered.

It is reasonable to assume that the interaction strength depended on the temporal proximity between the events in individual trials, being stronger when a saccade and a body movement occurred close together in time and negligible if they occurred far apart. Indeed, we found that the amplitude of the residuals depended on the time lag between the saccade and body movement onsets (***Fig 2A***), confirming the importance of considering lag-dependent interactions between the saccades and body movements in addition to the first-order terms [13]. To account for these observations and improve our estimates, we added a second-order term to the GLM as a convolution of a two-dimensional interaction kernel and a two-dimensional input (***Fig 2B***). Unlike first-order kernels, which span only one dimension, second-order (or interaction) kernels are quadratic and defined in two dimensions. Specifically, the two-dimensional factor *K*_*be*_ = W_be_(x_b_⊗x_e_) was computed considering the inputs x_b_ and x_e_, binary vectors of time lags from the onset of saccades and body movements (1 in the time bins where the saccade or body movement occurred and 0 otherwise) and W_be_, the interaction kernel, a ‘weighting’ element similar to first-order kernels W_b_ and W_e_, but computed for various x_b_ and x_e_ combinations of time lags, hence two-dimensional (***Fig 2B***). The interaction kernel (***Fig 2B***, *bottom*, example animal; ***S2 Fig***, population) quantified the difference between the first-order model’s prediction and the data for every point in time relative to both saccade and body movement onsets, computed in a ROI where the amplitude of the interaction was the largest.

When fitted to all mice (using ridge regularization), two main features were consistently observed in the interaction kernels. First, these kernels had a prominent region of suppression [13], particularly for pairs of events in which body movement preceded a saccade. Second, we noticed a high variability between the diagonals, that is, between slices of the kernel from the top left to the bottom right (e.g., in ***Fig 2B***, *bottom*). Since each diagonal corresponded to an interaction with a fixed relative lag between a saccade and a body movement, adjacent diagonals were computed from different trials; therefore, the variability between diagonals reflected not only the unavoidable variance of the kernel estimator due to the limited sample size (***S3 Fig***), but also the stochasticity of the neural responses across trials.

The variability between diagonals was also reflected in the elongated Fourier transform of the kernels (***Fig 2C***), which exhibited more power in the high frequencies in the top right and bottom left quadrants.

### Quantifying trial-by-trial variability via local regularization

We examined the link between the prominent region of suppression and trial-to-trial variability in the frequency domain. We hypothesized that the suppressive feature of the kernel is broad and slowly changing across lags but that the trial-to-trial variability—manifested in the variability between diagonals—affects it as a superimposed “noisy mask.” To quantify these components, we applied one of the most general and effective regularization techniques, ALD, which can be used to quantify the variability in interaction kernels via its optimal hyperparameters in the frequency domain [24, 28, 29]. Instead of equally penalizing all kernel weights, as in ridge regression, this regularization technique favors localized solutions with non-zero elements restricted to contiguous regions in the time and frequency domains. Priors determine the position, extent, and orientation of these regions in both domains, and their hyperparameters are optimized by ALD. Specifically, the hyperparameters *θ* are found by maximizing the log marginal likelihood *E(θ)* as follows:

$$E(\theta )=\log\;P(Y|X,\theta )=-\frac{n}{2}\log\left|2\pi \sigma^{2}\right|-\frac{1}{2}\log\left|C\Lambda^{-1}\right|+\frac{1}{2}\mu^{T}\Lambda \mu -\frac{1}{2\sigma^{2}}Y^{T}Y$$ (1)

where *X* is the input matrix, *Y* is a vector of observed values, *n* is the number of observations, *σ*^2^ is variance of noise, *C* is the prior covariance matrix, which depends on the parameters of the prior, $\Lambda ={\left(\frac{1}{\sigma^{2}}X^{T}X+C^{-1}\right)}^{-1}$ is the posterior covariance, and $\mu =\frac{1}{\sigma^{2}}\Lambda X^{T}Y$ is the posterior mean [24, 30]. The full set of parameters *θ* consists of *σ*^2^ and six parameters of *C*. The main difference between the temporal and the frequency domain consists in the prior covariance. Specifically, the prior covariance is modeled as a diagonal matrix in the temporal domain:

$$C_{ii}=\exp\left(-\frac{1}{2}{\left(\chi_{i}-\nu \right)}^{T}\Psi^{-1}(\chi_{i}-\nu )-\rho \right)$$ (2)

where *χ*_*i*_ is the location of the kernel weight *k*_*i*_ in the time lag coordinates of the two-dimensional interaction kernel, *ν* is the location of the center of the elliptical interaction region, Ψ is a 2 × 2 matrix determining the extent of the region, and *ρ* is the scale of the prior variance. On the other hand, in the frequency domain, the prior covariance is also modeled as a diagonal matrix as follows:

$$C_{ii}=\exp\left(-\frac{1}{2}{\left({|M\omega }_{i}|-\nu \right)}^{T}({|M\omega }_{i}|-\nu )-\rho \right)$$ (3)

where *ν* is the center of the elliptical region in the frequency domain, *ω*_*i*_ are the frequency coordinates, and $M=\left[\begin{array}{cc} M_{11} & M_{12}\\ M_{12} & M_{22} \end{array}\right]$ is a symmetric matrix.

**Fig 3:**
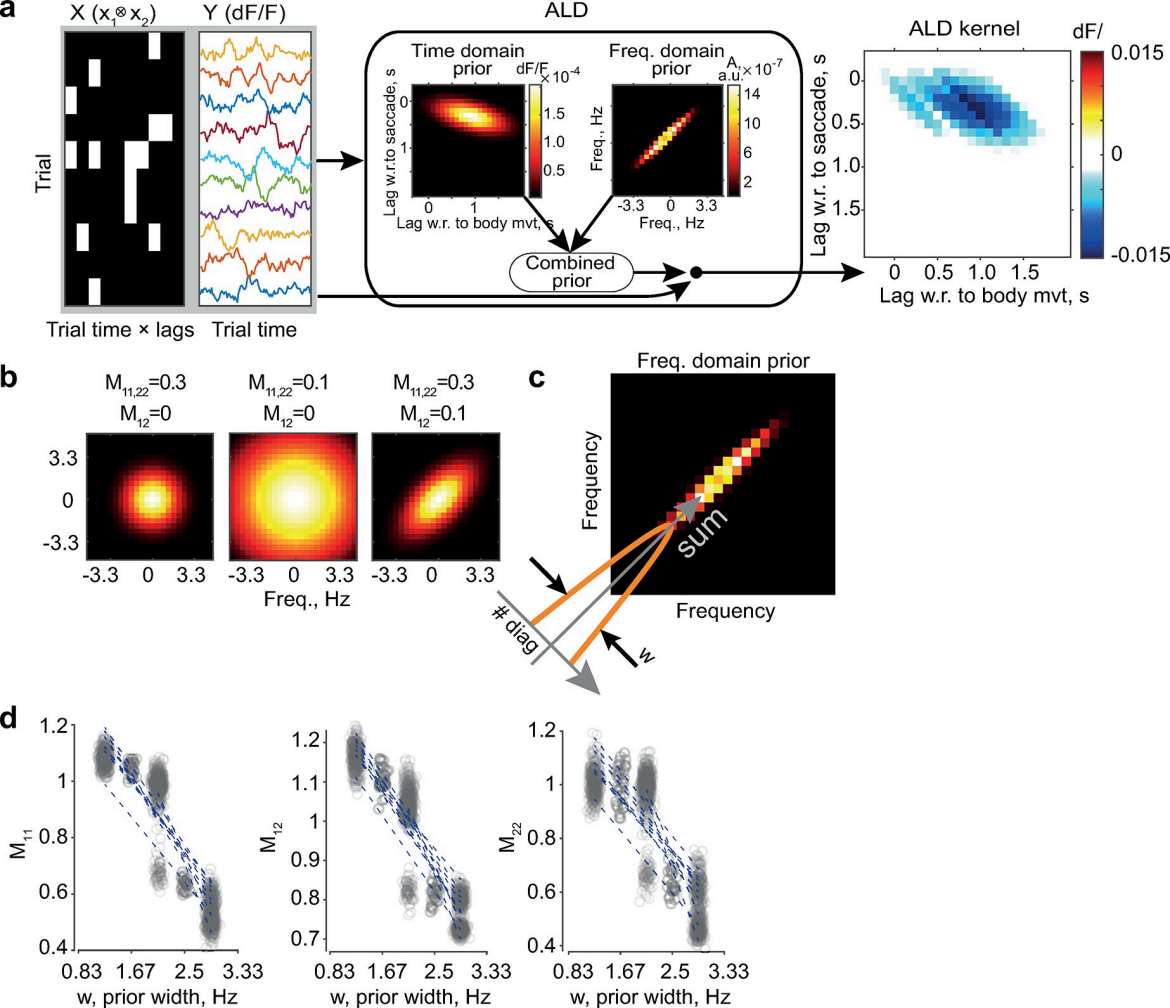
ALD hyperparameters capture frequency components in kernels that reflect trial-to-trial variability. (**a**) Schematic of ALD inputs and outputs. Left: input data: outer product of body movement and saccade event vectors (X) and dF/F residuals (Y) for every trial. Center: The ALD procedure finds time domain prior (left) and frequency domain prior (right, absolute value is shown), merges them, and computes a convolutional kernel by applying the merged prior to the data Y (long right-pointing arrow). Right: resulting kernel. Priors (center) and kernel (right) are shown for an example animal. (**b**) Frequency domain prior is parameterized with (M_11_, M_12_, M_22_), which makes up a symmetric matrix and which determines the shape and extent of the prior: diagonal (M_11_, M_22_) values regulate the extent (compare left and center), and off-diagonal terms (M_12_) increase the elongation. (**c**) Computation of the width *w* of the frequency domain prior. The absolute values in the frequency domain are summed along the diagonals (bottom left to top right), and the width is computed at 0.02 of the maximum value of the summed distribution (i.e., approximately the central 99.5% of the distribution for all animals). (**d**) The parameters *M*_*ij*_ of the ALD are inversely related to the width of the peak of the prior. One dot represents the *M*_*ij*_ and width values for a given animal. *M*_*ij*_ are fitted using hierarchical bootstrap: in every iteration (300 in total), sampling with replacement is done across mice and, for each animal, across trials. The correlations between widths and *M*_*ij*_ form all bootstrap iterations form a distribution whose values are compared to zero at a significance level of 0.05. The lines are example regression lines for 10 randomly selected bootstrap iterations. Five vertical stripes reflect the n = 5 mice in our dataset, with each animal having well-separated w-values at each iteration; the central stripe (mouse) has two distinct clouds representing two sets of *M*_*ij*_ fitting solutions.

Therefore, we re-fitted interaction kernels (still from the residuals of the first-order GLM) using this approach (Materials and Methods). We obtained localized and smooth estimates (***Fig 3A***, rightmost panel; example animal). Time and frequency domain priors (***Fig 3A***, *two center panels*; example animal) delineated the region of each kernel and its power spectrum. The frequency domain priors were oriented similarly to the power spectrum of the ridge regression kernel (***Fig 3A***, *center-right*; cf. ***Fig 2C***), stretching across the diagonals in the frequency domain, thus capturing the variability between the diagonals of the kernel in the time domain. Importantly, the shape of the prior in the frequency domain was quantified by a symmetric matrix *M*, similar to a Gaussian covariance matrix, with *M*_*12*_ regulating the elongation of the prior, and *M*_*11*_ and *M*_*22*_ determining the spread of the prior along the two coordinate axes (see equation for the prior covariance in the frequency domain above). Larger values of *M*_*11*_ and *M*_*22*_ corresponded to narrower priors (***Fig 3B,*** example frequency domain priors for several *M*_*ij*_ values; see also Materials and Methods). ALD favored elongated solutions parameterized with *M*_*12*_ > 0 (as in ***Fig 3A*** *center-right)*, observed in individual animals and across the population (*M*_*11*_ = 0.74 ± 0.24, *M*_*12*_ = 0.99 ± 0.24, *M*_*22*_ = 0.74 ± 0.19; mean ± SE).

The matrix *M* can provide a direct measure in the frequency domain of the trial-by-trial variability described above. To demonstrate this point, we examined the link between *M*_*ij*_ and the width of the priors *w* (***Fig 3C***, right; see Materials and Methods). We focused on width because it characterizes the extent to which the slowly changing suppressive feature of the kernels is masked by trial-to-trial variability. Concretely, an animal with low trial-to-trial variability and thus a relatively unmasked broad suppressive part of the kernel will have a wider frequency domain prior. We computed the width as the sum of the values in every diagonal along the orientation of the prior (***Fig 3C***). Across animals, *w* decreased with *M*_*ij*_ (Pearson’s r: r(*M*_*11*_,w) = -0.90 ± 0.11, p_boot_(r < 0) < 0.01; r(*M*_*12*_,w) = -0.92 ± 0.09, p_boot_(r < 0) < 0.01; r(*M*_*22*_,w) = -0.77 ± 0.31, p_boot_(r < 0) = 0.04; ***Fig 3D***). Although the hyperparameters *M*_*ij*_ correlated with the width of the priors, we found no significant correlation with the length of the priors computed in the direction perpendicular to *w*. The correlation found supports the conclusion that *M*_*ij*_ is a quantitative proxy of trial-by-trial variability. Further supporting evidence was found by examining the correlation between *M*_*ij*_ and the width of the Fourier transform of the ordinary least squares (OLS) kernels, with the FFT of the OLS kernels being a direct measure of the between- and along-diagonal variability of the temporal kernels. We found a tendency for a similar negative correlation, albeit not consistently significant (r(*M*_*11*_,w) = -0.61 ± 0.31, p_boot_(r < 0) = 0.046; r(*M*_*12*_,w) = -0.63 ± 0.32, p_boot_(r < 0) = 0.046; r(*M*_*22*_,w) = -0.56 ± 0.39, p_boot_(r < 0) = 0.103 n.s., ***S4 Fig***). A weaker significance was expected: although the width of both OLS and ALD priors is related to the suppressive feature of the kernel in the time domain, OLS kernels are generally noisier, since they are more prone to overfitting when trained using datasets of limited size [14].

In both previous analyses, we established significance using a hierarchical bootstrap [31]. We computed 300 sets of parameter estimates and width values and obtained a sampling distribution of correlations between *M*_*ij*_ and *w*, which we considered to be significantly below zero if the cumulative probability p_boot_ of r(*M*_*ij*_,w) > 0 was below the *α* = 0.05 criterion.

In summary, we first computed the interaction kernels using ALD, and then we found that across animals, the parameters *M*_*ij*_ decreased with an increasing width of the frequency domain priors, supporting the hypothesis that *M*_*ij*_ measures trial-by-trial variability. A similar qualitative trend between *M*_*ij*_ and the width of the Fourier transform amplitude was also found in the OLS kernels.

### Width of ALD frequency priors relates to the suppression strength in the kernels

The conclusion that *M*_*ij*_ is a quantitative proxy of trial-to-trial variability hinged on the observation that the width of the ALD frequency prior quantifies the masking of the slowly changing suppressive feature in the kernels caused by trial-to-trial variability. This observation, aside from being valid by “model design,” can be directly tested in our data. To do so, we first examined how the strength of the suppressive feature in the time domain depends on *M*_*ij*_−hence on the width of the ALD frequency prior (***Fig 3D***). We then manipulated the width of the power spectrum of an example kernel to causally examine changes in the suppressive features in the time domain.

**Fig 4:**
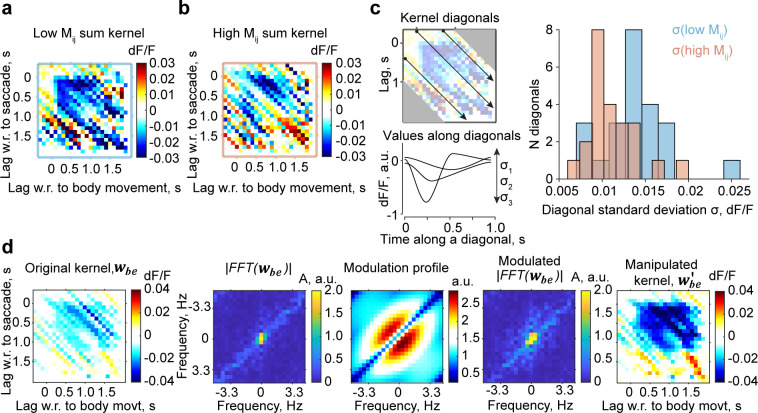
*M*_*ij*_ values and the width of ALD frequency prior quantify the trial-to-trial variability and the strength of the suppression in the time domain. (**a**) Pooled (summed) interaction kernel from two mice with lower *M*_*ij*_ values. (**b**) Summed interaction kernel of two mice with higher *M*_*ij*_ values. (**c**) Left: schematic of measurement of standard deviation of each kernel diagonal. Right: histogram of the standard deviation of kernel values along the diagonals in A (blue) and B (red) within a 1-s range of the main diagonal. (**d**) Broadening the power spectrum strengthens the kernel’s suppressive features. Left to right: (1) original kernel of an example mouse, (2) absolute value of its two-dimensional Fourier transform, (3) modulation profile designed to broaden the absolute value of the Fourier transform, (4) absolute value after modulation, and (5) manipulated kernel after an inverse two-dimensional Fourier transform.

Therefore, we first pooled the OLS kernels of mice with higher and lower *M*_*ij*_ separately (***Fig 4A and 4B***) and examined the variability (standard deviation) along each diagonal (***Fig 4C***, *right*). Grouping kernels according to *M*_*ij*_ values equates to pooling based on the width of the ALD frequency prior (***Fig 3D***). We found that the standard deviation was, on average, lower for mice with high *M*_*ij*_ than for mice with low *M*_*ij*_ (t-test, p = 0.032; ***Fig 4C***, left). This was due to the fact that kernels of mice with lower *M*_*ij*_ exhibited more prominent suppression immediately after the onset of either a saccade or body movement across a range of time lags. Similar profiles of the diagonals could be clearly observed in their average, which highlighted that the dF/F decrease after event onset and had sustained negative values for a more extended period (***S5 Fig***). This demonstrates the link between *M*_*ij*_, the width of the ALD frequency prior, and the strength of the kernel’s suppressive feature.

Second, for an example kernel obtained by ridge regression, we manipulated the absolute value of its Fourier transform by scaling up the amplitudes around the origin to increase the width of the power spectrum. This manipulation made the suppressive area of the kernel in the time domain more distinct than the original one, which became apparent after applying an inverse Fourier transform to the manipulated absolute values while maintaining the initial phases (***Fig 4D***). This effect was observed in the kernels of all mice. Together with the previous results, these observations indicate that the value of *M*_*ij*_, the width of the frequency domain prior, and the suppressive feature of the interaction kernel co-varied when describing animal-to-animal variability in saccade–body movement interactions.

### ALD hyperparameters correlate with changes in arousal

**Fig 5:**
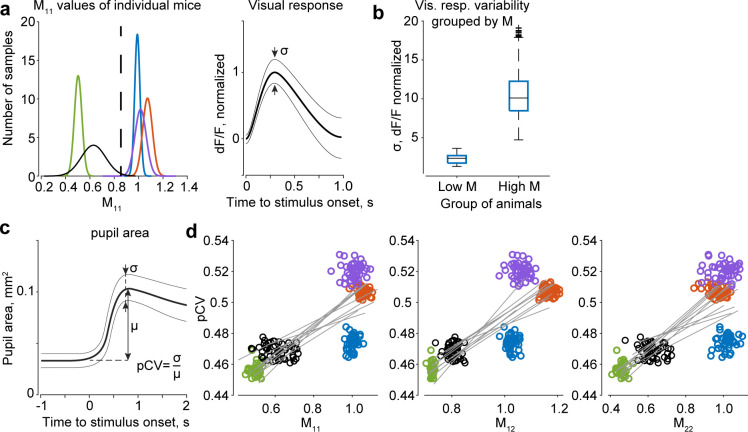
ALD hyperparameters correlate with changes in arousal gauged by the variability in visual responses and pupil area. (**a**) Left. Mice are classified as having high or low hyperparameter values (*M*_*11*_, *M*_*12*_, *M*_*22*_) relative to the population mean (black dashed line). Colored curves–smoothed distributions of bootstrap-mean *M*_*11*_ values of five animals. The distributions of *M*_*12*_ and *M*_*22*_ are positioned similarly. Right: schematic of the variability of normalized visual responses (σ, standard deviation). (**b**) Visual response variability is higher for animals with higher values of *M*_*ij*_ (x-axis: low-*M*/high-*M*, mice with *M*_*ij*_ below/above population average), p_boot_ < 0.001 (hierarchical bootstrap, n_B_ = 1000). Box plots: line–median, box– 25th and 75th percentiles; whiskers–full extent of the distribution; “+” symbols–outliers. (**c**) Calculation of pupil coefficient of variation (pCV) as a ratio of standard deviation of pupil area increase after the presentation of a visual stimulus to the mean pupil area increase. (**d**) Parameters *M*_*ij*_ correlate with the pupil coefficient of variation (pCV). *M*_*ij*_, pCV, and the corresponding correlations r(*M*_*ij*_,pCV) are computed using hierarchical bootstrap. In every iteration (n_B_ = 300), the significance of positive correlations is established by comparing the cumulative probability of r(*M*_*ij*_,pCV) < 0 to the 0.05 criterion (correlations are significant for all *M*_*ij*_). The data points corresponding to each mouse (n = 5) are indicated by different colors. The lines are regression lines for 10 randomly chosen bootstrap iterations.

Following results from one of our previous studies [13]—and in agreement with other works [32, 33]—we considered that the degree to which trial-to-trial variability in different animals affects the kernels and *M*_*ij*_ values might reflect more general variability in neural activity states between mice. To test this, we considered visually evoked responses used as an independent signal relative to eye and body movements. We correlated the variability of visually evoked responses (standard deviation of the peak response amplitude) with the *M*_*ij*_ parameters using trials in which visually evoked responses were not perturbed by movement-related activity (Methods). This set of trials did not overlap with the trials used in the analysis up to this point. The results showed that the activity evoked by the onset of the visual stimulus was indeed more variable in mice with higher *M*_*ij*_ (***Fig 5A and 5B***) (hierarchical bootstrap: p_boot_ < 0.001, n = 1000 bootstraps), suggesting that weak suppressive kernels (that is, those with larger *M*_*ij*_) were associated with higher trial-to-trial variability, even when such variability was estimated from stimulus-evoked components that were not included in the estimation of the interaction kernels.

Interestingly, differences in neural activity states between mice could be related to differences in the state of arousal or sustained attention as subjects engage in a behavioral task [34–36]. A typical biomarker of the level of arousal and sustained attention is the change in pupil area upon the presentation of a task-related stimulus [13, 37]. To quantify the differences in arousal levels between mice, we computed the pupil coefficient of variation (pCV, ***Fig 5C***) for each animal, defined as the standard deviation of the pupil area increase after stimulus presentation divided by its mean. This is an effective measure in this context because the average pupil size may vary due to factors that are not directly related to differences in states of arousal, such as body size. Remarkably, we found that the pCV indeed significantly and positively correlated with *M*_*ij*_ (r(*M*_*11*_,pCV) = 0.77 ± 0.23, p_boot_(r > 0) = 0.016; r(*M*_*12*_,pCV) = 0.80 ± 0.21, p_boot_(r > 0) = 0.013; p_boot_(r > 0) = 0.047, r(*M*_*22*_,pCV) = 0.70 ± 0.33; hierarchical bootstrap, n_B_ = 300 iterations; ***Fig 5D***), corroborating the hypothesis that trial-to-trial variability, as quantified by *M*_*ij*_, correlates with arousal states.

In summary, we found that *M*_*ij*_ changed along with the variability in neural activation, as indicated by the visual responses, and in states of arousal or sustained attention, as indicated by an independent metric derived from the variability in pupil area. Therefore, our results show that variability in states of arousal can affect the features of the interaction kernels and their frequency domain characteristics. As parameters *M*_*ij*_ quantify these characteristics, it can be concluded that these parameters reflect cognitive differences between animals, such as the ability to maintain steady engagement or attentional levels during a task and the degree to which frequent changes in motivation are typical for a given subject.

### Generalizing to other regularization methods

**Fig 6:**
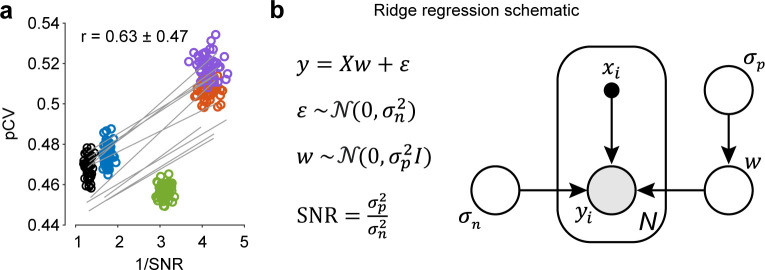
Correlation between ridge regression hyperparameters and pupil area variability. (**a**) Pupil area coefficient of variation (pCV, *Fig 5C*) versus signal-to-noise ratio (SNR) in hierarchical bootstrap samples. Colors correspond to different mice (n = 5); lines show linear regression on 10 example bootstrap iterations. The significance of positive correlations is established by comparing the cumulative probability of r(pCV, 1/SNR) < 0 to the 0.05 criterion (p_boot_(r > 0) = 0.08, n_B_ = 1000 HB). (**b**) SNR is computed as a ratio of prior variance $\sigma_{p}^{2}$ and noise variance $\sigma_{n}^{2}$ in a Bayesian formulation of ridge regression. $\sigma_{p}^{2}$ and $\sigma_{n}^{2}$ are found by evidence optimization. Left: linear regression model with Gaussian noise *ε*, weights *w*, and a ridge prior on these weights. Right: schematic of latent and observed variables in Bayesian ridge regression: deterministic inputs *x*_*i*_ and observations *y*_*i*_ in *i* = 1..*N* trials, latent kernel weights *w*, and latent standard deviations *σ*_*p*_ and *σ*_*n*_.

Finally, we investigated whether our results could be generalized to other regularization approaches. The original reasoning for choosing ALD regularization was to leverage locality information in time- and frequency-domain priors to gain interpretability of the response interactions. However, hyperparameters from other methods may be capable of quantifying the same variability, supporting the generality of our conclusion that regularization techniques may be used not only to prevent overfitting of the data but also to assess biologically relevant phenomena. For this purpose, we considered one of the most popular (but potentially less informative than ALD, in reference to “locality”) regularization techniques—ridge regression—and computed the inverse signal-to-noise ratio, 1/SNR = $\sigma_{n}^{2}/\sigma_{p}^{2}$, where $\sigma_{p}^{2}$ and $\sigma_{n}^{2}$ refer to prior variance and noise variance, respectively, in the Bayesian formulation of ridge regression (***Fig 6B***, see Methods). Given that 1/SNR captures the relative magnitude of noise and signal in every mouse, it is a good metric for assessing the difference in response variability across animals. First, we assessed whether the inverse of SNR from ridge and ALD hyperparameters *M*_*ij*_ correlated with each other. We found that, systematically across all animals, correlation values were positive for all *M*_*ij*_ hyperparameters, although not at a significance level *α* = 0.05 (r(*M*_*11*_, 1/SNR) = 0.43 ± 0.45, p_boot_(r > 0) = 0.15; r(*M*_*12*_, 1/SNR) = 0.48 ± 0.45, p_boot_(r > 0) = 0.12; r(*M*_*22*_, 1/SNR) = 0.30 ± 0.48, p_boot_(r > 0) = 0.24). It should be noted that p_boot_ provides a direct measure of probabilities relative to a particular hypothesis (contrary to typical summarized statistics, such as p-values [31]). Therefore, the result indicates that, systematically across mice, there was a high probability (~75%–90%) of finding positive correlations between *M*_*ij*_ and SNR. We then correlated the 1/SNR computed from ridge regression with the pupil CV (as done before), and found a systematic positive trend in correlations across animals (albeit again not at a significance level *α* = 0.05; r(pCV, 1/SNR) = 0.63 ± 0.47, p_boot_(r > 0) = 0.08, n = 1000 HB) (***Fig 6A***).

These results indicate that hyperparameters of “simpler” regularization methods, such as ridge regularization, could also be informative (although with higher p_boot_ probabilities, above a significant criterion alpha = 0.05) of underlying biological phenomena not included in the modeling framework, such as pCV. The observation that ALD produced more significant correlation values is not too surprising, given that with the prior knowledge about the parameters—their locality in time and frequency domains—ALD hyperparameters are better suited to characterizing variations in the underlying kernels, compared to the hyperparameters of ridge regression agnostic to the kernel structure. Together, these results suggest that the relationship between variability in states of arousal and sustained attention and model hyperparameters is likely to be found across different regularizing models.

## Discussion

In this study, we applied the ALD regularization technique to examine the interaction properties of neural signals associated with saccade and body movements in the posterior cortices of GCaMP-expressing mice. We found that across animals, ALD hyperparameters systematically changed with the amplitude of the interactions, with the variability in visually evoked responses, and with the overall trial-to-trial variability. We related this variability to fluctuations in the animals’ cognitive state—sustained attention and task engagement—as confirmed by a positive correlation between the ALD hyperparameters and variations in pupil size. Altogether, the ALD hyperparameters captured a systematic variation in the neural and cognitive states of the animals, providing information on factors that were not a priori included in the model’s design.

Despite the advantages of approaches based on spatial and spectral priors, such as ALD, we demonstrated that our findings can be generalized. Specifically, we showed that the ratio of noise variance and prior variance hyperparameters in ridge regression (i.e., the inverse of its SNR) also revealed a similar positive trend in the correlation with the pupil area as the one observed with the *M*_*i*,*j*_ hyperparameters characterizing the ALD spectral prior. We suggest that ALD more reliably captured the level of arousal with its hyperparameters, because it quantified the feature of the GLM interaction kernels that was most strongly affected by the response variability, namely the magnitude and the extent of the suppressive area of the kernel. On the contrary, hyperparameters of ridge regression—prior signal and noise variance—are not specific to periods of nonlinear interaction and ignore any underlying kernel structure, such as contiguous regions of suppression. The quantification of the variability of a particular response feature (as is the case for ALD hyperparameters), as opposed to the broad quantification of data variability (ridge hyperparameters), appears to be important for the significance with which the hyperparameters capture physiological phenomena, such as arousal in our case.

These observations cohere with the speculation that the more the model’s hyperparameters can account for “constraints” existing in the data, the more they can be informative about physiological phenomena not provided to the model. For instance, unlike general-purpose regularization procedures, such as ridge regression or automatic relevance determination [38], which impose agnostic constraints on parameter values, two-dimensional response kernel regularization can take advantage of a priori known spatial properties, such as smoothness [39–41], locality [24], and region-sparseness [42]. The ALD framework used in this study is typical of the latter class of models: it imposes local priors in time and frequency domains and quantifies them with hyperparameters. Whereas ridge regression hyperparameters quantify only the variance of noise and the prior variance of parameters, ALD hyperparameters quantify the position, extent, and orientation of a contiguous area in the time and frequency domains occupied by non-zero parameters. Due to this rich quantification, the optimal hyperparameter values can be compared between subjects and interpreted in the context of underlying physiological phenomena.

The dependence of regularizing hyperparameters on pCV is likely not unique to 2nd order interaction kernels. These kernels can be interpreted as a 2nd order linear expansion of a non-linear interaction process. We speculate that if a non-linear formalism also capable of including local priors is explicitly implemented, similar correlations might be found for the regularizing hyperparameters controlling the selected non-linearity.

Here, we showed that such hyperparameters could also reflect factors other than those that motivated their inclusion in the statistical model. In our data, the parameters *M*_*i*,*j*_ not only reflected the shape of the ALD frequency domain prior but also captured the variability in visually driven activations, the strength of the saccade–body movement interactions, and the variability in the animals’ state of arousal. The observed variability reflected both the stochastic nature of neural responses across trials and the unavoidable response variance due to the limited sample size. The latter effect became more pronounced as the number of trials in the subsampled datasets decreased. This demonstrates the advantage of regularizing models with parametric priors over more general methods and shows that hyperparameters can be informative of physiological correlates.

Although quantitatively ALD did not significantly improve cross-validated response predictions, it provided an advantage in explainability, with its hyperparameters quantifying the position, extent, and orientation of a contiguous area in the time and frequency domains occupied by non-zero parameters in the interaction kernels.

In this dataset, the regularizing hyperparameters can capture factors not explicitly included in the statistical model, significantly improving the estimation of 2D kernels given the response variability. A similar correlation between arousal and neural responses could be directly observed in the stimulus-evoked response and associated 1D kernels [13], which typically do not require regularization as they are less prone to overfitting.

The link between ALD hyperparameters and physiological properties, demonstrated in this study in the context of saccadic and body movements, and arousal, is likely to be found in other experimental preparations. Besides saccadic and body movements, two-dimensional response kernels (or “fields”) are often encountered, even within an individual sensory modality. For instance, when characterizing the properties of visual and auditory neurons, spatiotemporal and spectro-temporal receptive field estimations [43–47] routinely require regularization procedures due to the high number of parameters involved in their estimation [39–41].

Similarly, besides arousal, a broader range of factors could cause fluctuations in neural states both within and across behavioral sessions—for example, whether an animal’s behavior changes from goal-directed to habitual (e.g., as the animal becomes overtrained) or whether the choice strategy changes from relying on the stimulus or reward history to being history-independent. These sources of variability may not be captured by simply examining changes in pupil area, and likely demand more complex analyses (and monitoring) of animals’ behavior within and across trials [26, 48]. Dynamic state-space models that can link neural modulations to the pupil area, behavioral pattern shifts, and psychometric parameter changes [49, 50] represent a promising direction for future research.

Together, these considerations also underscore the importance of free-behavior paradigms and detailed and continuous monitoring of the animal’s behavioral state. In our data, a fundamental ingredient that gave rise to the variability across kernel diagonals—and, consequently, the ALD hyperparameters—was the variable time lags between eye and body movements. Due to this variability, adjacent kernel diagonals were pooled from trials that were typically not close together in time and were therefore associated with brain states (and neural response amplitudes) that varied within a training session. This event timing variability arose because the experimental design did not fully constrain the animals’ motor behavior. Although expert mice typically made movements after the onset of the visual stimulus, these movements could be considered uninstructed, since the animals made many body and eye movements within the response period (10-s duration) with no explicit go-cues. This may not be surprising since eye and body movement pairing is a natural motor pattern that occurs in the form of “orienting” movements in freely navigating mice and has also been indirectly observed in head-fixed mice [51]. The temporal order and latencies of these movement pairs may vary depending on an animal’s arousal level and motivation and may change along with the animal’s strategy of sampling its sensory environment. These observations emphasize the importance of free-behavior paradigms, which are more informative about a subject’s cognitive state and internal decision variables than the traditional controlled approach with rigidly timed trial epochs [52, 53]. Tracking uninstructed movements that are not relevant to completing a task can therefore prove helpful for explaining variance in neural [22] and behavioral observations [52] and can provide insights into animals’ heuristics. Indeed, in this study, the variability in states of arousal estimated through pupil dilation variability was central to interpreting the interaction kernels across animals.

Our findings are also likely generalizable in terms of the neural signals analyzed. In this study, we used wide-field fluorescence recordings, reflecting calcium-related cortical activity across multiple layers [23]. However, although these signals exhibit significant spatial correlations, the critical aspect of our results was that unique response signatures could be isolated, in both space and time, in reference to each movement type, which allowed the computation of the interaction kernels. Therefore, as long as this criterion is met with other signals, our formalisms are applicable, for example, to EEG or MEG data [54] or time-varying estimates of neurons’ firing rates.

Despite the interest and relevance of our results, it is important to consider some limitations in our conclusions, as the spatial and temporal characteristics of the analyzed signals impose significant constraints on the interpretability of our results. For instance, mesoscopic GCaMP fluorescence cannot provide access to the activity of individual neurons, thus limiting the interpretability of some features of the interaction kernels. It is unknown, for example, whether a negative interaction emerges due to mutual inhibition of the separate neural populations that encode body and eye movements. Alternatively, does this apparent interaction arise because the neural populations that encode body and eye movements partially overlap? Furthermore, the behavioral monitoring in our experiments was not exhaustive. Although we tracked major body movements associated with wheel turns, it has been reported that even the slightest “movement fidgets” can contribute to neural activation across layers and cell types [55]. Thus, a complete model of posterior cortical activation would also require more comprehensive monitoring of movement information at every moment during a trial.

In conclusion, our study shows that hyperparameters of regularization procedures can improve the fitting of multi-dimensional interaction kernels but can also provide relevant information on physiological processes that are not explicitly included in a model design. Because of these properties, hyperparameters can help improve current models by identifying factors that explain additional variance in the data and that are most often not explicitly included in the models’ formalisms.

## Materials and methods

### Ethics statement

All surgical and experimental procedures were approved by the Support Unit for Animal Resources Development of RIKEN CBS (W2022-2-47).

### Animals and task

The data used in this study were first presented in ref. [13], where a more detailed description of the methods was provided. We trained Thy1-GCaMP6f mice (n = 5) daily in a 2AFC orientation discrimination task using automatic cages featuring self–head fixation [25]. After reaching a 75% success rate, the mice were used in imaging experiments in which they performed the same task. For all reported results, the number of sessions per animal ranged from 20 to 64, with the trials per animal ranging from ~3000 to ~8000. We included only animals whose ALD parameter values were stable across bootstrap resamples of the data.

### Behavioral training

The animals were trained in a 2AFC orientation discrimination task. Two oriented Gabor patches (20° static sinusoidal gratings, sf = 0.08 cpd, randomized spatial phase, two-dimensional Gaussian window, sigma = 1/4 of the patch diameter) were shown on the left and right sides of a screen in front of the animal (LCD monitor ProLite B2776HDS-B1, Iiyama, Japan; 33.6 cm × 59.8 cm [~58° × 100° dva], 1080 × 1920 pixels, 25-cm distance from the animal) at ±35° eccentricity relative to the body’s midline. The mice had to determine which of the two stimuli matched a target orientation (vertical or horizontal, fixed for each animal).

The animals indicated their choices by rotating a rubber wheel with their front paws (***Fig 1A***), which synchronously shifted the stimuli on the screen horizontally [25, 56]. When the correct target was shifted to the center of the screen, the animals were rewarded with 4 μL of water. Incorrect responses were discouraged with a prolonged (10-s) inter-trial interval (ITI) and a flickering checkerboard stimulus (2 Hz). If no response was made within 10 s (time-out trials), neither reward nor discouragement was given. Once in training, the animals could obtain water only by making correct choices in the task and received agarose as a top-up if their performance was worse than necessary for weight maintenance.

Each trial consisted of an open-loop (OL) period (1.5 s), in which the wheel manipulator was decoupled from the stimuli, and a closed-loop (CL) period (0–10 s), in which the wheel changed the position of the stimuli. The CL period was followed by an ITI (3–5 s, randomized). Stimuli were presented throughout the OL and CL periods. We recorded cortical responses, wheel rotations, and eye-tracking videos from a 1-s pre-stimulus period until the end of each trial. Only data until the end of the OL period were used.

### Psychometric curve

We fitted the animals’ probability of making a right-side choice as a function of task difficulty using a psychometric function ψ(Δθ;α,β,γ,λ)=γ+(1−γ−λ)F(Δθ;α,β), where *F*(*x*) is a Gaussian cumulative probability function, *α* and *β* are the mean and standard deviation, respectively, *γ* and *λ* are the left and right lapse rates, respectively, and Δ*θ* is the difference in the angular distance to the vertical orientation, calculated as Δ*θ* = |*θ*_*L*_|−|*θ*_*R*_|. Confidence intervals were computed using bootstrapping (n = 999). The population psychometric curve was computed as the average of the curves of the individual mice.

### Behavioral readouts

#### Eye tracking

We monitored the left contralateral eye illuminated by an IR LED (SLS-0208-B medium beam; Mightex), using a CMOS camera (FL3-U3-13E4M-C; Point Grey) equipped with a zoom lens (Navitar Zoom 7000; 1280 × 1024 pixels; typical ROI size: 350 × 250 pixels; 30-Hz acquisition rate) with an IR filter (Kenko PRO1D R72; 52 mm). The camera was aligned to the perpendicular bisector of the eye, forming an approximately 60° angle with the midsagittal axis of the animal. The pupil position was automatically tracked using custom software (MATLAB; MathWorks). We detected saccadic eye movements by applying an adaptive elliptic thresholding algorithm to the XY velocities of filtered XY positions in the pupil center [13].

We obtained the pupil area by converting the pixels of eye-tracking videos to millimeters using direct measurements of the width of the eye to account for experiment-to-experiment camera zoom variability. We considered a relative pupil area measure by subtracting the average pupil area of a given session from all of the trials of that session. We characterized each trial with the respective pupil area increase, computed as the difference between the pupil area at the moment of stimulus presentation and the maximum pupil area within the OL period.

### Wheel rotations

We considered wheel rotation onset an instance in which the wheel changed the direction of rotation, and eventually rotated 20°. Smaller movements were considered unintentional and discarded. Wheel rotations were generally associated with full-body movements, often including hind limb, tail, trunk, and facial movements. Thus, we considered wheel rotation onsets as instances of detected body movements.

### Imaging

During the experiments, expert mice were placed under a macroscope for wide-field imaging (THT; Brain Vision), with the head fixed using a head plate latching system [25]. We used a tandem-lens epifluorescence macroscope equipped with a CMOS camera (pco.edge 5.5; 5.5 MP; pixel size: 6.5 μm^2^) and two lenses (NIKKOR, Nikon; 50 mm, F1.2, NA = 0.46) to image GCaMP6f fluorescent signals: excitation light, 465-nm LED (LEX2B; Brain Vision); emission filter, band-pass at 525 ± 25 nm (Edmund).

We recorded GCaMP fluorescence of the posterior part of the dorsal cortex of the right hemisphere through a cranial window (5 mm diameter) centered on the primary visual cortex. The cranial window spanned all the higher visual areas and parts of the retrosplenial and somatosensory areas.

### Retinotopy

We computed retinotopic maps with a standard frequency-based method in which slowly moving horizontal and vertical flickering bars were presented to an anesthetized animal (~0.8% isoflurane). We performed visual area segmentation based on azimuth and elevation gradient inversions [57–59]. We centered and oriented the maps across animals using the centroid of area V1 and the iso-azimuth line passing through it [57].

### Preprocessing wide-field GCaMP6f signals

We motion-corrected the GCaMP data and registered all image frames to a previously acquired retinotopic map using the control point selection method (MATLAB). We computed the relative fluorescence responses (dF/F) by first calculating the grand average scalar as ${F_{0}}^{i,j}=<I_{x,y,t}^{i,j}{>}_{x,y,t}$, where $I_{x,y,t}^{i,j}$is the XYT image tensor in trial *i* of session *j*, and then normalizing the raw data tensor to that value as $F_{x,y,t}^{i,j}=\left(I_{x,y,t}^{i,j}-{F_{0}}^{i,j}\right)/{F_{0}}^{i,j}$. The data in each trial were then band-pass-filtered (0.1–12 Hz) and smoothed using mild spatial filtering (Gaussian σ = 20 μm). We then compressed each tensor using spatial binning (130 × 130 μm^2^ with 50% overlap). The presented results did not critically depend on any of these parameters.

### Data modeling

We baseline-corrected each trial used in modeling to the average dF/F within −1 to −0.8 s before stimulus onset. We discarded any trials with saccades or body movements during this interval.

### Generalized linear model

We fitted the GLM to dF/F GCaMP responses that were downsampled to 10 Hz and baseline-corrected by subtracting the mean relative activation dF/F in the -1 to -0.8-s interval before stimulus onset. At any given trial, we modeled the GCaMP response as $y(t)=\sum_{i=1}^{I}w_{i}*x_{i}+\varepsilon$, where *w*_*i*_ represent the convolution kernels, *ε* is Gaussian noise, and *x*_*i*_ are the event vectors of inputs. The model accounted for three individual inputs—stimulus onset, eye movement (saccade), and body movement—and a two-dimensional input of interacting saccades and body movements. Event vector *x*_*i*_ had 1’s in the time bins of the events and 0’s in all other time bins. We computed the interaction input as an outer product of the corresponding *x*_*i*_. The convolution kernels *w*_*i*_ acted both causally and anti-causally, accounting for both pre- and post-movement/saccade responses. Since the data were baseline-corrected, no bias term was introduced. Given that the stimulus co-occurred with saccades and body movements, kernels fitted using trials with multiple events would suffer from multicollinearity.

To minimize the resulting trade-off in kernel values, we fitted them in a sequence. First, we fitted the stimulus kernel using responses in trials in which no saccade or body movement was detected. The kernel contained both the stimulus response and the average baseline before and after it in a window of -1 to 1.5 s, unperturbed by any other events. Next, we subtracted this kernel from all the trials and used the residuals to compute the saccade and body movement kernels. In each case, we used a set of trials in which a single saccade or a single body movement was sufficiently temporally isolated from all other events. We fitted all three kernels using regularized least squares with ridge regularization, in which the regularization hyperparameter was automatically determined through evidence maximization. We estimated the interaction kernels from the residuals dF/F after subtracting the stimulus, body movement, and saccade responses. We estimated the interaction kernels using OLS, ridge regression, and ALD, initially with no normalization and using units of dF/F. OLS kernels were computed using the normal equation $\hat{w}={\left(X^{T}X\right)}^{-1}X^{T}y$, where *X* is a design matrix of *x* with rows corresponding to time bins of *y* and columns corresponding to kernel dimensions *i*.

The ridge-regularized kernels of stimulus, saccade, and body movement responses shown in ***Fig 1C and 1E*** were baseline-corrected to dF/F at -100 ms relative to stimulus onset and -300 ms relative to saccade and movement onsets, normalized by the peak dF/F at the ROI of the respective response, spatially aligned, and averaged across animals. For presentation purposes, the averages were filtered using a 20 × 20-pixel-wide mean filter (the size of the activation map in ***Fig 1*** is approximately 160 × 160 pixels). The time traces in ***Fig 1E*** are across-animal averages based on each animal’s response in the corresponding ROI (region of maximum dF/F for the respective event within or close to retinotopically identified areas). ROIs for each event were computed as follows: we first identified the time of peak response amplitude and then selected pixels above a varying threshold, from 70th to 99th percentile at steps of 0.5 percentiles, to create binary mask images. We then averaged the masks and defined an ROI as a contiguous group of pixels above the 99th percentile.

An example interaction kernel (***Fig 2B***) is shown in units normalized to the stimulus response amplitude in the stimulus ROI and corresponds to the tile (part of the brain surface) where the interaction is strongest. A value in the kernel was masked if it was estimated from fewer than 10 trials.

### Automatic locality determination

Wide-field GCaMP responses associated with movements are temporally localized (i.e., they are triggered at the time of movement and finished within a finite time window), and we can expect that the interactions of these responses are also temporally localized. The temporal locality of an interaction response translates into a locality of non-zero weights of the two-dimensional interaction kernels (***Fig 2B***, *bottom*) and can be formalized as a prior that penalizes kernel weights that are far from the temporal region of interaction. ALD finds these priors in the temporal domain for the kernel and in the frequency domain for the Fourier transform of the kernel. The priors are parametric: the local region of interaction is assumed to have an elliptic shape, and the parameters of the prior quantify its extent and orientation. The parameters *θ* are found by maximizing the log marginal likelihood *E(θ)* as follows:

$$E(\theta )=\log\;P(Y|X,\theta )=-\frac{n}{2}\log\left|2\pi \sigma^{2}\right|-\frac{1}{2}\log\left|C\Lambda^{-1}\right|+\frac{1}{2}\mu^{T}\Lambda \mu -\frac{1}{2\sigma^{2}}Y^{T}Y$$ (4)

where *X* is the input matrix, *Y* is a vector of observed values, *n* is the number of observations, *σ*^2^ is variance of noise, *C* is the prior covariance matrix, which depends on the parameters of the prior, $\Lambda ={\left(\frac{1}{\sigma^{2}}X^{T}X+C^{-1}\right)}^{-1}$ is the posterior covariance, and $\mu =\frac{1}{\sigma^{2}}\Lambda X^{T}Y$ is the posterior mean [24, 30]. The full set of parameters *θ* consists of *σ*^2^ and six parameters of *C*, as described below. Optimization is first performed separately in the temporal and frequency domains, after which the two optimal covariance matrices are combined [24]. In this study, we primarily focused on the parameters of the frequency domain prior.

In the temporal domain, the prior covariance is modeled as a diagonal matrix as follows:

$$C_{ii}=\exp\left(-\frac{1}{2}{\left(\chi_{i}-\nu \right)}^{T}\Psi^{-1}(\chi_{i}-\nu )-\rho \right)$$ (5)

where *χ*_*i*_ is the location of the kernel weight *k*_*i*_ in the time lag coordinates of the two-dimensional interaction kernel, *ν* is the location of the center of the elliptical interaction region, Ψ is a 2 × 2 matrix determining the extent of the region, and *ρ* is the scale of the prior variance. The parameters defining covariance are thus three in Ψ, two in *ν*, and a one-dimensional *ρ*.

In the frequency domain, the prior covariance is modeled as a diagonal matrix as follows:

$$C_{ii}=\exp\left(-\frac{1}{2}{\left({|M\omega }_{i}|-\nu \right)}^{T}({|M\omega }_{i}|-\nu )-\rho \right)$$ (6)

where *ν* is the center of the elliptical region in the frequency domain, *ω*_*i*_ are the frequency coordinates, and $M=\left[\begin{array}{cc} M_{11} & M_{12}\\ M_{12} & M_{22} \end{array}\right]$ is a symmetric matrix. This matrix is similar to a covariance matrix of a Gaussian probability distribution in that off-diagonal elements regulate the elongation of the elliptical region in frequency coordinates, and diagonal elements regulate its extent, but it is different in that there is no requirement for positive definiteness; thus, for example, values *M*_*12*_ can be greater than *M*_*11*_ and *M*_*22*_. The hyperparameters in this case are two in *ν*, three in *M*, and *ρ*. After the parameters are optimized, the interaction kernel weights in the time and frequency domains are computed as a mode of the posterior.

Finally, the two covariance matrices—*C*_*t*_ for time domain covariance and *C*_*f*_ for frequency domain covariance—are combined for a time–frequency prior as follows:

$$C=C_{s}^{2}(B^{T}\;C_{f}\;B)C_{s}^{2}$$ (7)

In this study, we focused on the link between the matrix *M* and the shape of the frequency domain prior. Therefore, to isolate the effect of *M* (***Figs 3D***, ***5D*** and ***S4***), we initially fitted all the parameters of the time and frequency domain priors, set all parameter values except *M*_11_, *M*_12_, and *M*_22_ to the population averages, and fitted the models for individual mice again. We also found that additional parameter constraints were necessary for the initial fits to be reasonable. For the temporal domain prior to be compact and within the early time lags of the kernel, it was sufficient to set the correlation coefficient parameter in Ψ to 0.5 (which meant the diagonal orientation of the interaction area) and *σ*^2^ to 0.001 for both the frequency and time domain priors (which was greater than the unconstrained optimum by less than one order of magnitude for most animals).

### Analysis of ALD parameters and hierarchical bootstrap

#### Hierarchical bootstrap

We used a hierarchical bootstrap to obtain the distributions of the correlations between *M*_*ij*_ and the width of the frequency domain prior (***Fig 3D***), between *M*_*ij*_ and the width of the Fourier transform of the OLS kernels (***S4 Fig***), and between *M*_*ij*_ and the pCV (***Fig 5D***), as well as the distribution of the responses to the visual stimulus pooled across low- and high-*M* animals (***Fig 5B***). The hierarchical bootstrap accounts for the differences in the amount of available data from different animals and for within-animal dependencies that can change from mouse-to-mouse. It involves two-level bootstrap sampling (with replacement): sampling of animals and sampling of trials of these animals [31]. For correlation analyses, in every hierarchical bootstrap iteration (total n_B_ = 300 iterations), we sampled with replacement n_A_ = 5 animals. For each of them, we sampled their trials using bootstrap n_T_ = 20 times. For each of the n_T_ trial samples, we fitted the interaction kernels using ALD (***Figs 3D*** and ***5D***) and OLS (***S4 Fig***). Thus, we had [n_B_, n_A_, n_T_] sets of parameters of ALD and an equal number of OLS kernels. We also computed the pCV from the same resampled trials shown in ***Fig 5D***. We averaged the obtained *M*_*ij*_ parameters and kernels across n_T_ for each fixed bootstrap and animal iteration, obtaining n_A_ = 5 sets of parameters for each iteration. We next removed the outlier iterations based on the values of *M*_*11*_, *M*_*12*_, *M*_*22*_ and pCV, computed the correlations between the parameters of interest—*M*_*ij*_ and prior width (***Fig 3D***), width of the Fourier transform of the n_T_-averaged OLS kernel (***S4 Fig***), and *M*_*ij*_ and pCV (***Fig 5D***)—obtained the distribution of correlations across bootstraps, and determined whether this distribution was significantly above or below zero. We constructed the frequency domain priors in this procedure using the n_T_ averages of the parameter values obtained from the hierarchical bootstrap. We considered the correlations to be significantly negative if the 95th percentile of the distribution of the correlation coefficients lay below 0. We followed a similar procedure for the amplitudes of the responses to visual stimuli shown in ***Fig 5B***: after we categorized animals as either low- or high-*M* (relative to the population mean), we sampled one animal in each group, for which we sampled trials with visual stimulus responses (n_B_ = 1000 times in total).

### Kernel Fourier transform width

To compute the width of the kernels in the frequency domain, we first computed the average value of each anti-diagonal (those oriented from bottom left to top right for a kernel transform, as shown, for example, in ***Fig 2C***). We subtracted the smallest value from all diagonal averages and computed the width at the level of 0.15 × maximum for the Fourier transform of OLS and at the level of 0.02 × maximum for the frequency domain prior. We chose levels below half height because both the priors and the Fourier transforms of OLS kernels were quite narrow and similar at half height.

### Analysis of kernels with high and low *M*_*ij*_

For the pooled kernels of low- or high-*M* animals (***Fig 4A and 4B***), we used OLS kernels fitted using hierarchical bootstrap. For every mouse, we averaged the kernels from all bootstrap iterations. We then summed the kernels of the two mice with the lowest *M*_*ij*_ values and the kernels of the two mice with the highest *M*_*ij*_ values separately. To examine the variability for a fixed lag between a body movement and a saccade, we computed the standard deviation along each diagonal of the kernel for lags between -800 and 800 ms.

### Analysis of SNR in ridge regression models

In the Bayesian approach to ridge regression [30], two hyperparameters are optimized: prior variance of parameters, $\sigma_{p}^{2}$, and variance of noise (e.g., measurement errors), $\sigma_{n}^{2}$. Based on estimates of these hyperparameters, we computed SNR as a ratio $\sigma_{p}^{2}/\sigma_{n}^{2}$.

### Kernel manipulation by widening the power spectrum

For the enhancement of the suppressive region of the kernel shown in ***Fig 4D***, we broadened the power spectrum of an example animal by multiplying its absolute value by an arbitrary profile (***Fig 4D***, center) that was a product of a lognormal function (starting from the main anti-diagonal and moving along the diagonals in both directions) and a 1/x function of distance from the center of the power spectrum. After multiplying the absolute value of the Fourier transform, we summed the result with the original phase and performed an inverse Fourier transform to obtain the kernel with enhanced suppression, as shown in the rightmost panel.

## Supporting information

S1 Fig Body movement response with or without correction for hemodynamics. (PDF)

S2 Fig Interaction kernels of the five mice used in this study. (PDF)

S3 Fig Variability in the estimates of the hyperparameters due to sample size. (PDF)

S4 Fig The width of the Fourier transform of the OLS kernel estimates negatively correlates with the M_ij_ values. (PDF)

S5 Fig Mean of the diagonals of the pooled kernel shown in Fig 4. (PDF)
