# Supplementary material for: Regularizing hyperparameters of interacting neural signals in the mouse cortex reflect states of arousal
Source: PLoS Comput Biol. 2024 Oct 15;20(10):e1012478. doi: 10.1371/journal.pcbi.1012478 (PMC11527387; doi:10.1371/journal.pcbi.1012478)
Supplement: S1 Fig — (PDF) [file pcbi.1012478.s001.pdf]

Supplementary Figures

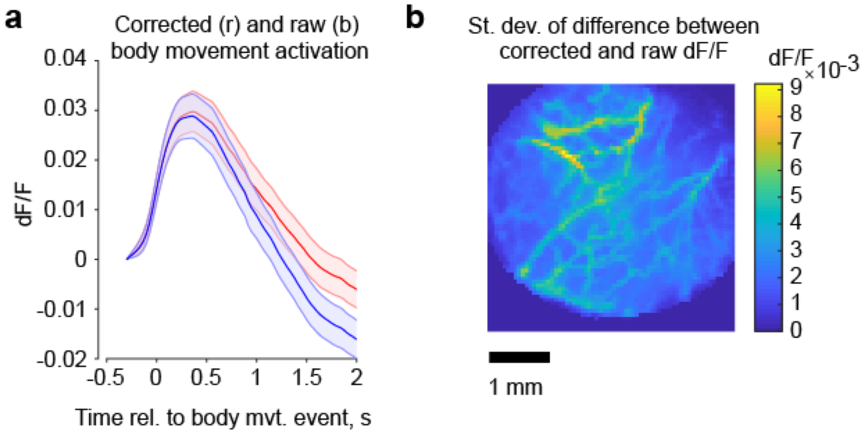

Supplementary Figure 1. Body movement response with or without correction for hemodynamics. (**a**) Average response to a body movement in the region (tile) of maximum response. Blue: single-wavelength dF/F signal; red: dF/F signal corrected for hemodynamics. The period of the strongest saccade–body movement interaction (see the Interaction Kernels subsection) is in time windows of up to 1 s relative to a body movement, when the effect of the hemodynamic correction is small. (**b**) Standard deviation of the difference between the corrected and uncorrected average body movement responses in the time interval of −0.3 to 2 s around the body movement onset. Blood vessels are more affected by hemodynamic correction.
