# Supplementary material for: Regularizing hyperparameters of interacting neural signals in the mouse cortex reflect states of arousal
Source: PLoS Comput Biol. 2024 Oct 15;20(10):e1012478. doi: 10.1371/journal.pcbi.1012478 (PMC11527387; doi:10.1371/journal.pcbi.1012478)
Supplement: S2 Fig — (PDF) [file pcbi.1012478.s002.pdf]

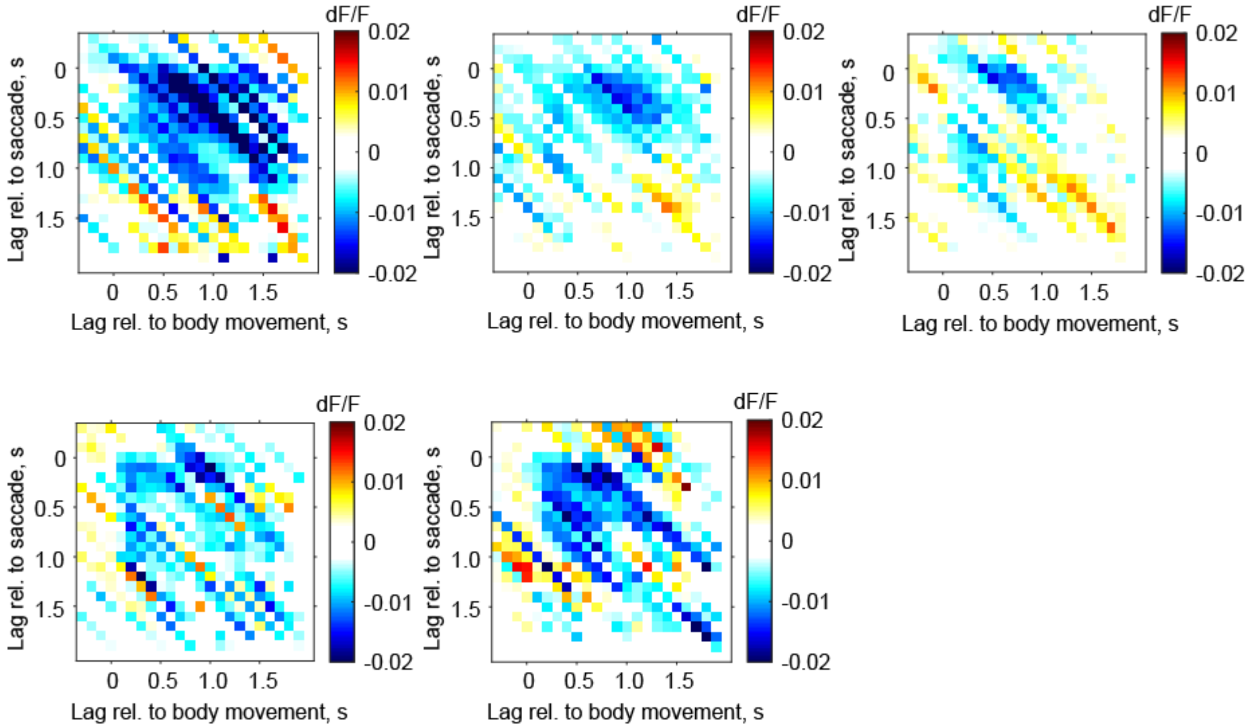

Supplementary Figure 2. Interaction kernels of the five mice used in this study: ridge regression estimate.
