# Supplementary material for: Regularizing hyperparameters of interacting neural signals in the mouse cortex reflect states of arousal
Source: PLoS Comput Biol. 2024 Oct 15;20(10):e1012478. doi: 10.1371/journal.pcbi.1012478 (PMC11527387; doi:10.1371/journal.pcbi.1012478)
Supplement: S3 Fig — (PDF) [file pcbi.1012478.s003.pdf]

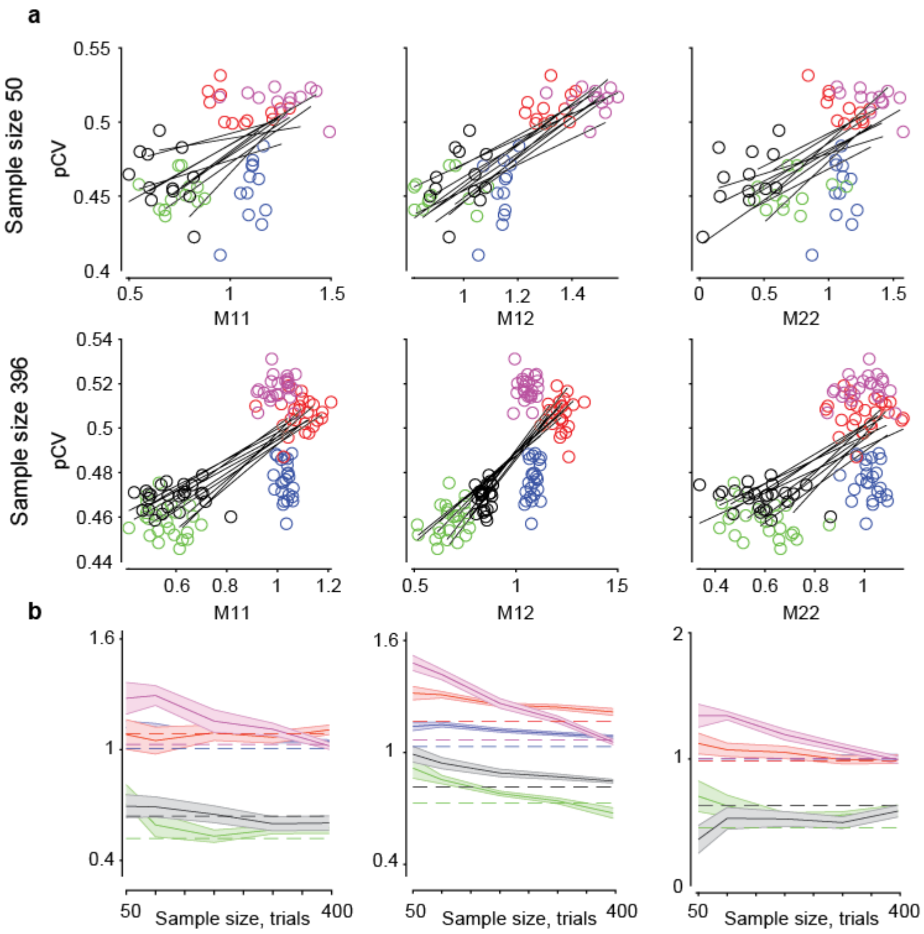

Supplementary Figure 3. Variability in the estimates of the hyperparameters due to sample size**.** (**a**) Similar analysis as in Figure 5, subsampling the dataset by considering only 50 trials for each animal (top 3 panels), to 396, that is, the smallest of the total number of trials across all mice (bottom 3 panels); columns are for different hyperparameters (x-labels). (**b**) Dependence of M_{ij} (y-axis, top labels) on the number of trials. Different colors are for different animals; shaded band are 95% confidence intervals. Dashed lines of the same color are M_{ij} values for the corresponding mouse using all available trials.
