# Supplementary material for: Regularizing hyperparameters of interacting neural signals in the mouse cortex reflect states of arousal
Source: PLoS Comput Biol. 2024 Oct 15;20(10):e1012478. doi: 10.1371/journal.pcbi.1012478 (PMC11527387; doi:10.1371/journal.pcbi.1012478)
Supplement: S4 Fig — (PDF) [file pcbi.1012478.s004.pdf]

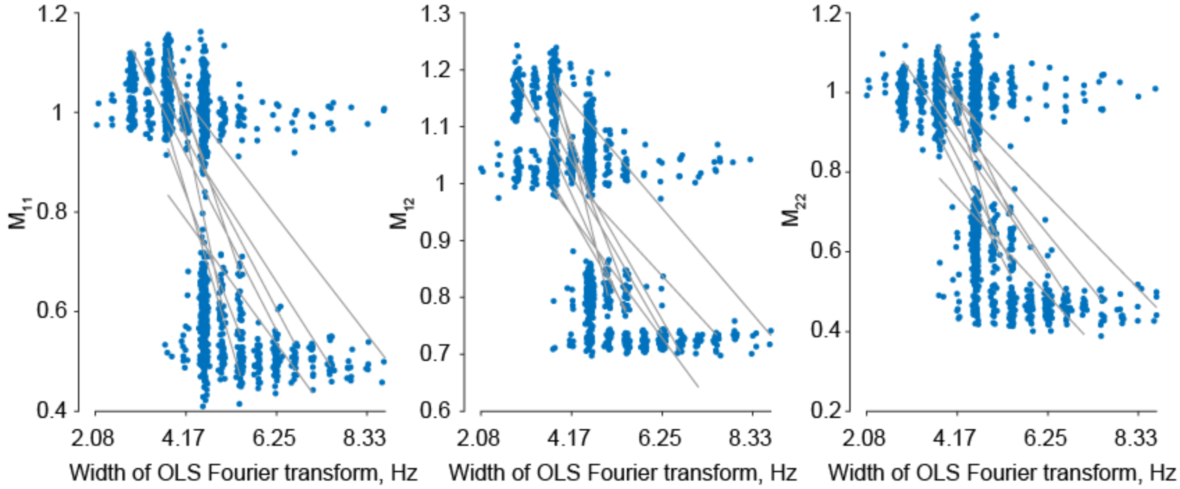

Supplementary Figure 4. The width of the Fourier transform of the OLS kernel estimates negatively correlates with the M_{ij} values. The OLS kernels and ALD parameters M_{ij} are fitted using trials selected through hierarchical bootstrapping. In every bootstrap iteration, five mice are sampled using bootstrap, and trials of every animal are sampled using bootstrap. Pearson's correlation coefficient is computed for every bootstrap iteration. Whether the correlation is significantly negative is assessed according to the sample distribution of correlations (criterion α = 0.05). The lines are regression lines randomly selected for 10 bootstrap iterations. The widths are computed according to the schematic shown in ***Figure 3c***, at a "height" equal to 0.15 of the maximum value of the sum.
