# Supplementary material for: Regularizing hyperparameters of interacting neural signals in the mouse cortex reflect states of arousal
Source: PLoS Comput Biol. 2024 Oct 15;20(10):e1012478. doi: 10.1371/journal.pcbi.1012478 (PMC11527387; doi:10.1371/journal.pcbi.1012478)
Supplement: S5 Fig — (PDF) [file pcbi.1012478.s005.pdf]

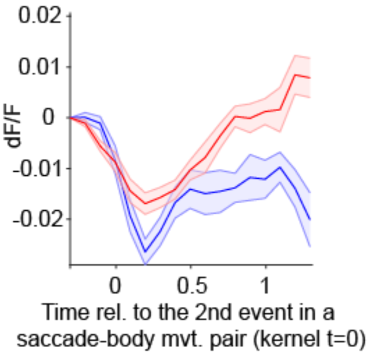

Supplementary Figure 5. Mean of the diagonals of the pooled kernel shown in ***Figure 4a*** (blue) and *4**b*** (red) for the range of diagonals (relative lags between saccade and body movement) from −800 to 800 ms.
